# Long-Term High Salt Intake Involves Reduced SK Currents and Increased Excitability of PVN Neurons with Projections to the Rostral Ventrolateral Medulla in Rats

**DOI:** 10.1155/2017/7282834

**Published:** 2017-12-06

**Authors:** Andrew D. Chapp, Renjun Wang, Zixi (Jack) Cheng, Zhiying Shan, Qing-Hui Chen

**Affiliations:** ^1^Department of Kinesiology and Integrative Physiology, Michigan Technological University, Houghton, MI 49931, USA; ^2^Department of Biotechnology, School of Life Science, Jilin Normal University, Siping, Jilin 136000, China; ^3^Biomolecular Science Center, Burnett School of Biomedical Sciences, College of Medicine, University of Central Florida, Orlando, FL 32816, USA

## Abstract

Evidence indicates that high salt (HS) intake activates presympathetic paraventricular nucleus (PVN) neurons, which contributes to sympathoexcitation of salt-sensitive hypertension. The present study determined whether 5 weeks of HS (2% NaCl) intake alters the small conductance Ca^2+^-activated potassium channel (SK) current in presympathetic PVN neurons and whether this change affects the neuronal excitability. In whole-cell voltage-clamp recordings, HS-treated rats had significantly decreased SK currents compared to rats with normal salt (NS, 0.4% NaCl) intake in PVN neurons. The sensitivity of PVN neuronal excitability in response to current injections was greater in HS group compared to NS controls. The SK channel blocker apamin augmented the neuronal excitability in both groups but had less effect on the sensitivity of the neuronal excitability in HS group compared to NS controls. In the HS group, the interspike interval (ISI) was significantly shorter than that in NS controls. Apamin significantly shortened the ISI in NS controls but had less effect in the HS group. This data suggests that HS intake reduces SK currents, which contributes to increased PVN neuronal excitability at least in part through a decrease in spike frequency adaptation and may be a precursor to the development of salt-sensitive hypertension.

## 1. Introduction

Elevated sympathetic outflow is a characteristic of salt-sensitive hypertension (SSH). The increased sympathetic outflow elicited by elevated dietary consumption of sodium chloride (NaCl) has been well documented to underlie the neural mechanisms of SSH, although many other stressors contribute to the development of SSH. Recent reports from our lab [1–4] and others [5–8] have demonstrated that the development of SSH is at least in part, neurogenic in nature. Indeed, numerous studies have demonstrated a link between elevated extracellular NaCl and the development of exaggerated sympathetic outflow [2, 9–12] as well as central hyperosmotic NaCl challenge consistently increases SNA [2, 9–14]. However, how HS intake activates the sympathetic nervous system (SNS) neurogenically has not been well studied.

While there are many areas in the brain that contribute to the sympathetic activation and development of SSH, the paraventricular nucleus (PVN) of the hypothalamus has numerous projections to downstream brain regions such as the nucleus solitary tract [15], rostral ventrolateral medulla [3, 4] (RVLM), and spinal cord [16, 17]. These downstream brain regions are capable of regulating and/or initiating sympathetic outflow which contributes to hypertension. A key pathway in the neuronal circuitry for the development of hypertension is neurons originating in the PVN which have a monosynaptic projection to the RVLM (PVN-RVLM) [1, 3, 4]. The RVLM has pathway projecting to the spinal intermediolateral column (IML) [18] which has high regulatory control on sympathoexcitation. As such, any alterations in PVN-RVLM neuronal excitability can have a profound effect on the sympathoexcitation.

Like other central nervous system (CNS) regions, PVN neuronal activity is regulated not only by synaptic activity but also by intrinsic membrane properties and excitability. Small conductance Ca^2+^-activated K^+^ (SK) channels have been documented in brain regions as a major regulator of neuronal excitability [19–22]. It has been demonstrated that the SK channels also play an important role in controlling the *in vitro* excitability of presympathetic PVN-RVLM neurons and *in vivo* sympathetic outflow in rats [3, 23]. Moreover, we have reported that downregulation of SK channel function among the PVN neurons contributed to the sympathoexcitation in rats with chronic HS intake (5 weeks of 2% NaCl) [24]. Furthermore, our recent study indicates that depletion of endoplasmic reticulum (ER) Ca^2+^ store likely plays a role in increasing PVN-RVLM neuronal excitability, which may underlie the mechanisms of sympathoexcitation in rats with HS intake [24]. Due to the fact that Ca^2+^ release from the ER is a prominent activator of SK channels to mediate neuronal excitability [25], we hypothesize that reduced SK currents may contribute to the increased excitability of PVN-RVLM neurons in rats with HS intake, which may underlie the neural mechanism of sympathoexcitation. This in turn may be a precursor and contributing factor to the development of SSH through alterations of the intrinsic properties of presympathetic PVN-RVLM neurons.

## 2. Methods

### 2.1. Animal Preparation and High Salt Diet

Male Sprague-Dawley rats (*n* = 21, 250–400 gm, Taconic) were individually housed in a temperature-controlled room (22-23°C) with a 14 h : 10 h light-dark cycle. Rats were age matched and placed on either high salt (HS, 2% NaCl) or normal salt (NS, 0.4% NaCl). Diets were identical in calories from fat and protein and also total carbohydrate and sucrose (Harlan). All experimental and surgical procedures were carried out under the guidelines of the National Institutes of Health Guide for the Care and Use of Laboratory Animals with approval by the Institutional Animal Care and Use Committee of Michigan Technological University.

### 2.2. Retrograde Labeling of PVN-RVLM Neurons

**Figure 1:**
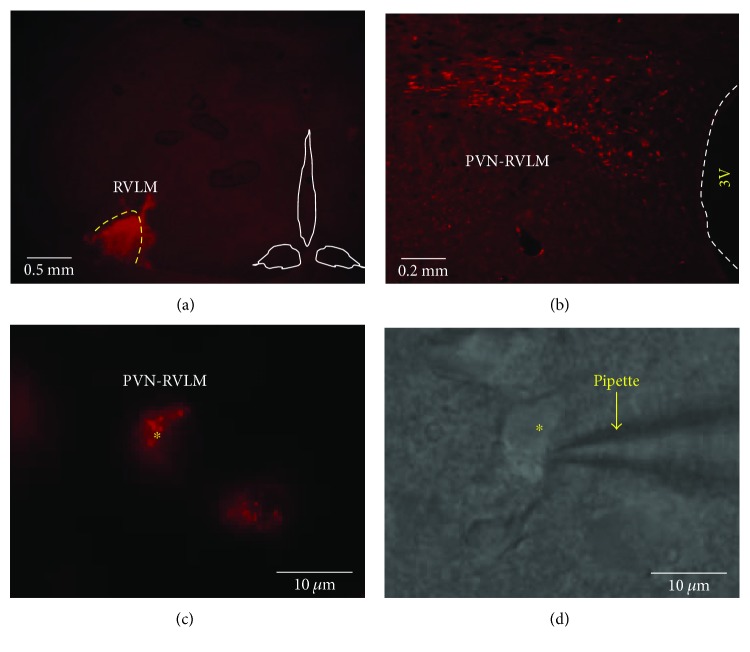
Identification of PVN-RVLM neurons in brain slice for whole-cell patch clamp recording. (a) Red fluorescent tracers were microinjected (100 nL) into the RVLM. (b) Retrograde labeling of the ipsilateral PVN was observed in the dorsal and ventrolateral subnuclei. (c) Image viewed with fluorescence illumination shows a recorded PVN-RVLM neuron containing retrograde tracer. (d) Infrared-DIC image shows the patch electrode positioned on the same PVN-RVLM neuron as (c). 3V: third ventricle.

Rats on HS diet were labeled after 5 weeks of HS feed, and NS rats were age matched and labeled as needed. PVN neurons were retrogradely labeled from the ipsilateral RVLM as previously described [1, 3, 16]. Briefly, rats were anesthetized with intraperitoneal sodium pentobarbital (50 mg/kg body weight) and placed in a stereotaxic frame, and a small burr hole drilled to expose the cerebellum. A glass micropipette was lowered into the pressor region of the RVLM (coordinates: −13.0 mm caudal to bregma, 1.8 mm lateral to midline, and 9.2 mm below the skull) and 100 nL of red fluosphere microspheres (Life Technologies) injected. Each animal received daily injections of penicillin G (30,000 U/100 gm body weight, subcutaneously) and meloxicam (1 mg/kg body weight) for 3 days after surgery. Location of the tracer was verified postmortem in histological sections through the RVLM (Figure 1). Identical surgeries were performed on rats on a NS diet.

### 2.3. Electrophysiology

To perform whole-cell patch clamp recordings, methods utilized were adapted from our and others' previous publications [1, 4, 26]. Briefly, five to seven days following retrograde labeling, rats were anesthetized with isoflurane (5% in O_2_) and decapitated. Brains were rapidly removed and submerged in ice-cold cutting solution (~3 min) containing (in mM) 206 sucrose, 2 KCl, 2 MgSO_4_, 1.25 NaH_2_PO_4_, 26 NaHCO_3_, 1 CaCL_2_, 1 MgCl_2_, 10 D-glucose, and 0.4 ascorbic acid. Osmolality and pH were adjusted to 290–302 mosmol/L and 7.32–7.40, respectively. Cutting solution pH and pO_2_ were maintained by continuous gassing with 95% O_2_–5% CO_2_. A brain block containing the hypothalamus was cut and fixed on a vibrating microtome (Leica VT 1000S; Leica, Nussloch, Germany). Coronal slices through the PVN were cut at a thickness of 250 *μ*m. Slices were incubated at 30°C for 1 h in artificial cerebrospinal fluid (ACSF) containing (in mM) 125 NaCl, 2 KCl, 2 MgSO_4_, 1.25 NaH_2_PO_4_, 26 NaHCO_3_, 2 CaCl_2_, 10 D-glucose, and 0.4 ascorbic acid (osmolality: 295–302 mosmol/L, pH 7.32–7.40). Slices were transferred to a glass-bottomed recording chamber and viewed through an upright microscope (Nikon) equipped with DIC optics, epifluorescence, an infrared (IR) filter, and an IR-sensitive video camera (C2400, Hamamatsu, Bridgewater, NJ). An appropriate filter was used to visualize neurons retrogradely labeled with the red fluospheres (Figure 1).

Patch electrodes were pulled (Flaming/Brown P-97, Sutter Instrument, Novato, CA) from borosilicate glass capillaries and polished to a tip resistance of 4–8 MΩ as previously described [1, 4, 26]. Electrodes were filled with a solution containing (in mM) 135 K-gluconate, 10 HEPES, 0.1 EGTA, 1.0 MgCl_2_, 1.0 NaCl, 2.0 Na_2_ATP, and 0.5 Na_2_GTP (osmolality: 280–285 mosmol/L, pH 7.3). Our intracellular solution contains a low concentration of EGTA (0.1 mM) to allow the accumulation of intracellular Ca^2+^ during membrane depolarization for the activation of SK channels [3, 16]. Once a GΩ seal was achieved in whole-cell configuration, cell capacitance (*C*_m_), access resistance, and resting membrane potential (*V*_m_) were monitored until stable. Cells that met the following criteria were included in the analysis: action potential amplitude ≥50 mV from threshold to peak, input resistance (*R*_input_) ≥ 0.5 GΩ (determined by injection of −20 pA from a holding potential of −80 mV), resting *V*_m_ negative to −50 mV. Recordings were made using an Axopatch 200B amplifier and pCLAMP software (10, Axon Instruments, Union City, CA). Signals were filtered at 1 kHz, digitized at 10 kHz (Digidata 1400A, Axon Instruments), and saved on a computer for off-line analysis.

### 2.4. Recording SK Current

To study SK current, voltage-clamp recordings were performed as previously described [1, 4], with intracellular solution containing a cAMP analogue 8-(4-chlorophenylthio) 3,5′-cyclic adenosine monophosphate (8CPT-cAMP, 50 *μ*M) to block the slow afterhyperpolarization current [1]. Recordings were performed in the presence of tetrodotoxin (TTX, 1.0 *μ*M) to block voltage-gated sodium channels and tetraethylammonium (TEA, 1.0 mM) to block voltage-gated potassium channels. Membrane potential was clamped at −60 mV and stepped to +10 mV for 100 ms. On returning *V*_m_ to −60 mV, an outward tail current was recorded. A time control was performed at 5, 10, and 15 min. After time control tail currents, apamin (100 nM) was bath applied to selectively block SK channels and again recorded at 5, 10, and 15 min postapamin treatment. The tail current recorded during treatment was subtracted from the corresponding control tail current to isolate the apamin-sensitive SK current. Decay of the SK current was analyzed by fitting the subtracted current with a one-phase exponential.

### 2.5. Testing Neuronal Excitability

Excitability of neurons from NS and HS rats was studied in a current-clamp mode in the absence of TTX, TEA, or 8CPT-cAMP as was previously described [1, 4, 26], with slight modification. With membrane potential adjusted to −80 mV by continuous negative current injection, a series of square-wave current injections was delivered in steps of +25 pA, each for a duration of 800 ms. To determine the action potential voltage threshold (*V*_t_), ramp current injections (0.2 pA ms^−1^, 1000 ms) were made from a potential of −80 mV. Square wave and ramp current injections were made in the same neurons. For the depolarizing *R*_input_, a +25 pA current injection was made for 800 ms and the voltage change measured. The depolarizing *R*_input_ was calculated using Ohms' law (*V* = (*I*/*R*)). It must be noted that current and voltage-clamp recordings were made from different groups of PVN-RVLM neurons.

### 2.6. Chemicals

All chemicals were obtained from Sigma-Aldrich (St Louis, MO, USA) except for TTX (Tocris Bioscience, UK) and TEA (Fluka BioChemika, Switzerland).

### 2.7. Data Analysis

Summary data are reported as means ± SEM. Depending on the experiment, group means were compared using an unpaired test, a one-way or a two-way ANOVA with post hoc analysis. When differences were found, the Newman-Keuls multiple comparison test was used for comparison test (GraphPad Prism, v5.0). Differences between means were considered significant at *P* < 0.05.

## 3. Results

### 3.1. Comparison of Passive Membrane Properties

Resting membrane potential (*V*_m_), depolarizing *R*_input_, whole-cell capacitance (*C*_m_), and voltage threshold for firing action potentials (*V*_t_) of PVN-RVLM neurons from NS controls and the HS group were compared, and no significant differences were identified either in the absence or presence of bath application of apamin (Table 1).

### 3.2. High Salt Diet Reduces SK Currents in PVN-RVLM Neurons

**Figure 2:**
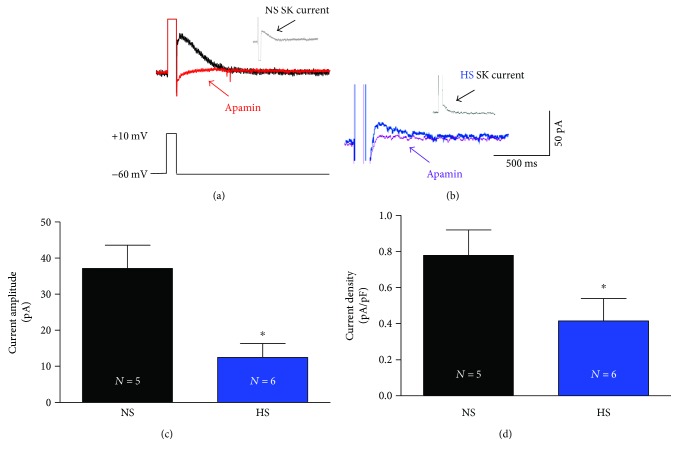
HS intake reduces SK currents in PVN-RVLM neurons. (a) The representative voltage-clamp recording traces from NS PVN-RVLM neuron. An outward tail current (black) prior to apamin and the outward tail current (red) after apamin with the total SK current (inset) from the subtraction of the apamin trace (red) from the nonapamin trace (black). (b) The representative voltage-clamp recording traces from HS PVN-RVLM neuron. An outward tail current (blue) prior to apamin and the outward tail current (purple) after apamin with the total SK current (inset) from the subtraction of the apamin trace (purple) from the nonapamin trace (blue). (c) Summary data for apamin-sensitive SK currents between NS controls and the HS group. (d) Summary data for SK current density between NS controls and the HS group showed a significant reduction in SK current density (^∗^*P* < 0.05 versus NS controls, unpaired *t*-test).

Our lab has previously demonstrated that HS diet and subcutaneous infusion of ang II induce a diseased state model of SSH in male SD rats. This hypertensive model shows reduced SK currents and increased PVN-RVLM neuronal excitability [1]. To determine whether HS alone is capable of altering PVN-RVLM SK currents, we performed whole-cell voltage-clamp recordings of SK currents in PVN-RVLM neurons from NS and HS diet-treated SD rats at a holding potential of −60 mV. Our results show that SK currents in HS rats were significantly reduced compared to NS rats (12.9 ± 6.9 pA versus 37.1 ± 6.5 pA, *P* < 0.05).  Figure 2(a) shows a representative SK current trace from the same neuron of before apamin, a SK channel blocker in NS (black), after treatment of apamin in NS (red), and the subtracted total SK current in NS (*inset*).  Figure 2(b) shows a representative SK current trace from the same neuron in HS rats, before apamin (blue), after apamin (purple), and the subtracted total SK current (*inset*).  Figure 2(c) is summary data of SK currents in NS (black, *n* = 5) and HS (blue, *n* = 6).  Figure 2(d) is summary data for SK current density between NS (black) and HS (blue). The SK current density was calculated by the total SK current divided by the cell capacitance.

### 3.3. High Salt Intake and Role of SK Channels in Regulating Neuronal Excitability

**Figure 3:**
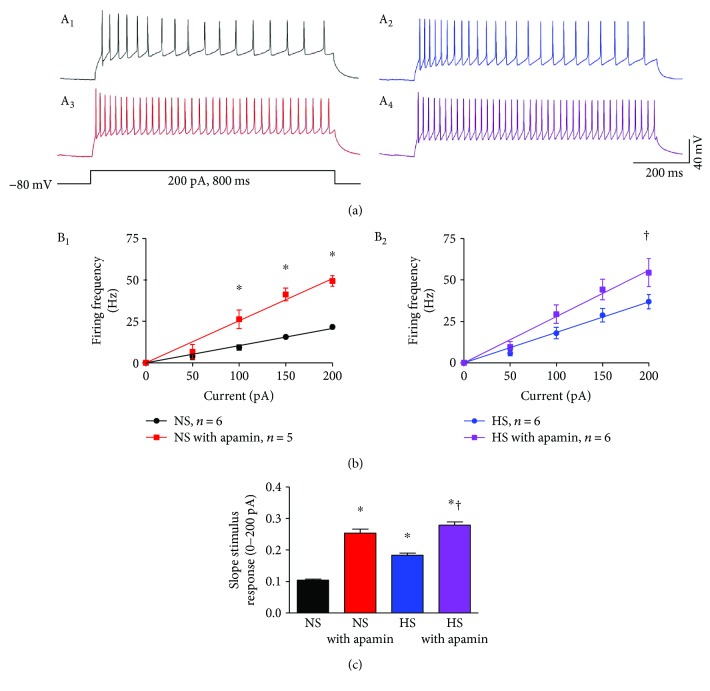
HS intake increases PVN-RVLM neuronal excitability. (a, black) A representative trace of NS control neuronal firing at +200 pA current injection. (a, blue) A representative trace of HS control neuronal firing at +200 pA current injection. (a, red) A representative trace of neuronal firing at +200 pA current injection in NS with apamin. (a, purple) A representative trace of neuronal firing at +200 pA current injection in HS with apamin. (b, left) A current injection stimulus-response curve for NS controls (black) and NS with apamin (red) to graded current injections (^∗^*P* < 0.05 versus NS controls without apamin, two-way ANOVA). (b, right) A current injection stimulus-response curve for the HS group (blue) and HS with apamin (purple) to graded current injections (†*P* < 0.05 versus the HS group without apamin, two-way ANOVA). (c) Summary data for the slope of the linear regression line from the current injection response. The HS group showed a significant increase in the slope compared to NS controls. Treatment with apamin significantly increased the slope in both NS controls and the HS group, but less effect on the slope of the HS group compared to NS controls. (^∗^*P* < 0.05 versus NS controls; †*P* < 0.05 versus the HS group, one-way ANOVA).

To test whether HS intake alters excitability and whether reduced SK currents underlie the mechanisms of HS intake induced an increase in excitability, PVN-RVLM neurons from NS and HS-treated rats were subjected to sequential depolarizing current injections (0–200 pA, positive). Graded current injections evoked graded increases in firing frequency and reached saturation at +200 pA current in NS neurons. This data confirms our previous findings regarding PVN-RVLM neuronal excitability between NS and HS-treated rats [24]. Apamin, a potent SK channel blocker, significantly increased neuronal firing frequency in NS neurons compared to NS neurons without apamin (+200 pA, 49.4 ± 3.2 versus 18.5 ± 2.5 Hz, *P* < 0.05).  Figure 3 shows representative traces of NS control (Figure 3(a), black), NS with apamin (Figure 3(a), red), HS control (Figure 3(a), blue), and HS with apamin (Figure 3(a), purple) to +200 pA current injection. A current injection stimulus response was constructed between NS and NS with apamin over the sequential depolarizing current injections (0–200 pA). Significant increases (^∗^*P* < 0.05) in excitability were observed at 100, 150, and 200 pA current injection in NS with apamin (Figure 3(b), left, red line, two-way ANOVA) compared to NS control (Figure 3(b), left, black line, two-way ANOVA). A linear regression of the current injection response was well fit and showed an increased slope of the line in NS with apamin compared to NS control (Figure 3(c), 0.25 ± 0.013 versus 0.10 ± 0.004, ^∗^*P* < 0.05 versus NS). Likewise, PVN-RVLM neurons from HS rats showed a significant increase in firing frequency compared to NS neurons (30.0 ± 4.8 versus 18.5 ± 2.5 Hz, *P* < 0.05). SK channel blockade with apamin in HS PVN-RVLM neurons increased neuronal excitability compared to HS control (45.4 ± 5.4 versus 30.0 ± 4.8 Hz, *P* < 0.05) but did not show a difference when compared to NS with apamin (45.4 ± 5.4 versus 49.4 ± 3.2 Hz). A current injection stimulus-response curve was constructed for HS control and HS with apamin PVN-RVLM neurons over the depolarizing current injections (0–200 pA). A significant (†*P* < 0.05) difference in excitability was noticed at 200 pA current injection in HS control (Figure 3(b), right, blue line, two-way ANOVA) compared to HS with apamin (Figure 3(b), right, purple line, two-way ANOVA). A linear regression of the current injection response was well fit and showed an increased slope of the line in HS with apamin compared to HS control (Figure 3(c), 0.28 ± 0.010 versus 0.18 ± 0.007, †*P* < 0.05 versus HS). Treatment of NS and HS with apamin increased the slope of the line almost identically, (0.25 ± 0.013 versus 0.28 ± 0.010) which were not statistically different. This data suggests that SK channels contribute to the regulation of neuronal excitability and that HS reduces SK channel function which contributes to increased PVN-RVLM neuronal excitability through an increase in sensitivity to a stimulus.

### 3.4. High Salt Intake and Role of SK Channels in Regulating Spike Frequency Adaptation

**Figure 4:**
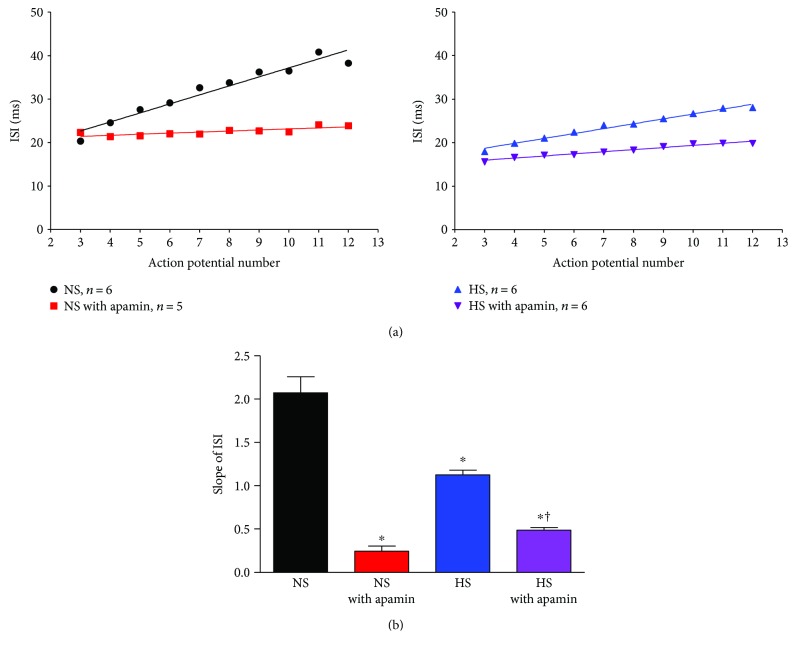
HS intake decreases interspike interval (ISI) in PVN-RVLM neurons. (a, left) ISI time for NS controls (black) and NS with apamin (red) PVN-RVLM neurons. (a, right) ISI time for the HS group (blue) and HS with apamin (purple) PVN-RVLM neurons. (b) Summary data for the slope of the linear regression line for the ISI in NS controls and NS with apamin, ISI in the HS group and HS with apamin (^∗^*P* < 0.05 versus NS; †*P* < 0.05, versus HS, one-way ANOVA).

To test whether dysfunction of SK channels contributes to the increased neuronal excitability in rats with HS intake through the inhibition of spike frequency adaptation (SFA), PVN-RVLM neurons from NS and HS, with and without apamin, were analyzed for interspike interval time (ISI) on action potentials 2–12 at +200 pA current injection. We found that compared to NS control cells, blockade with apamin significantly reduced the ISI time (Figure 4(a), left). Furthermore, HS cells without apamin also exhibited a decreased ISI time compared to NS control cells (Figure 4(a), right). The ISI lines were fitted with a linear regression and the slopes were analyzed to give an understanding of the overall SFA. Compared to NS control cells, NS with apamin had a significantly reduced slope of the line (Figure 4(b), 2.07 ± 0.186 versus 0.242 ± 0.058, ^∗^*P* < 0.05 versus NS). Similarly, HS control cells compared to HS with apamin had a significantly reduced slope, although not as drastic as in the NS cells (Figure 4(b), 1.23 ± 0.055 versus 0.485 ± 0.033, †*P* < 0.05 versus HS). HS control cells compared to NS control cells showed a significant decrease in the slope of the line (1.23 ± 0.055 versus 2.07 ± 0.186, ^∗^*P* < 0.05 versus NS). NS and HS cells treated with apamin had very similar slopes for ISI, (0.242 ± 0.058 versus 0.485 ± 0.033) which were drastically reduced compared to cells without apamin. This data suggests reduced SK channel function in PVN-RVLM neurons contributes to the increased excitability in rats with HS intake through the mechanism of inhibition of spike frequency adaptation (SFA).

## 4. Discussion

The present study explored the effects of high salt (HS) intake on SK currents and the role of SK channels in regulating excitability among presympathetic PVN neurons. The major findings of this study were (1) the amplitude of whole-cell SK current was reduced and neuronal excitability was increased in the HS group compared with NS controls. SK channel blockade with apamin induced a greater increase in excitability in NS controls compared to that in the HS group; (2) A shorter interspike interval (ISI) was observed in the HS group compared to NS controls. Apamin significantly shortened the ISI in NS controls but had less effect in the HS group. This data suggests that HS diet reduces SK currents, which contributes to increased PVN-RVLM neuronal excitability at least in part through an increased sensitivity to a stimulus and a decrease in spike frequency adaptation (SFA).

We have previously demonstrated that rats with ang II-salt hypertension have reduced SK currents and increased PVN-RVLM neuronal excitability [1, 27, 28]. The effect of HS intake alone on the properties and function of SK channels in these neurons have not been well studied yet. What is clear is that chronic HS intake is capable of altering many neuronal properties. Previous publications regarding chronic HS intake have implicated a number of ion channels and neuronal signaling mechanisms which are affected, including downregulation of the potassium/chloride cotransporter 2 in vasopressin neurons [29], increased proinflammatory cytokine production [26, 30], increased NOX [31–34], reduced GABA [35–37] levels, and reduction in glutamate decarboxylase-67 in the PVN [33]. These studies provide direct evidence that chronic HS intake does have a dramatic effect on neuronal signaling molecules and on neuronal excitability. Despite this fact, very little is known regarding the effects of HS intake on PVN-RVLM neuronal excitability and SK currents in normotensive rats. That is to say, we were interested in whether SK channel dysfunction is secondary to hypertension or whether SK channel dysfunction precedes the development of hypertension.

How SK currents are reduced in rats with a HS diet is a bit unclear at this point. It is unlikely that the alterations are due to increases in sodium concentrations in the cerebral spinal fluid (CSF). Nakamura and colleagues have demonstrated that SD rats on a HS diet have no observable changes in sodium concentration in CSF compared to animals with NS control [38]. Furthermore, the PVN is encapsulated by a complete blood-brain barrier (BBB) indicating that influx of peripheral hormones is less likely [39]. We cannot however rule out upstream circumventricular organs (CVO) such as the subfornical organ (SFO), which have an incomplete BBB and are sensitive to changes in osmolality and circulating peripheral hormones such as Ang II [11, 27, 40, 41]. The SFO does have a projection to the PVN, and it has been postulated that alterations in SFO-PVN-RVLM neurons may contribute to the development of hypertension [11, 41]. Whether this presynaptic input of CVOs is capable of altering SK channel function through changes in presynaptic neurotransmitter release or gene expression remains to be elucidated.

Contrary to the unknown mechanisms of HS intake-induced increases in the excitability of presympathetic PVN neurons, the contributions of SK channels in regulating neuronal excitability in other areas of the brain have been consistently documented in the literature. In nonhypertensive studies, SK channels have been shown to modulate the excitability of neurons in the infralimbic cortex, contributing to fear extinction memory [42]. Similar to our studies in PVN-RVLM neurons, Criado-Marrero and colleagues showed an increase in neuronal excitability following blockade of SK channels with apamin, a decrease in ISI time and an increase in depolarizing *R*_input_ in the infralimbic cortex [42]. In a separate study, Yang found that SK channels in the dorsal horn of the spinal cord also controlled neuronal excitability and mAHP, with potential therapeutic value for treatment of pain [22]. In cardiac motor neurons, Lin and colleagues also established SK channel blockade with apamin-increased neuronal firing frequency, decreased mAHP, and abolished spike frequency adaptation (SFA) compared to neurons treated without apamin [43]. These groups contribute to the well-established concept that SK channels significantly regulate neuronal excitability, both in autonomic sympathetic neurons, parasympathetic neurons, and cortical neurons. Our conclusions from autonomic PVN-RVLM neurons are also consistent with these previous findings. It must also be mentioned that there is some regional specificity regarding SK channel activity and neuronal excitability. Gu and colleagues have reported differences in SK channel regulation of neuronal excitability between CA1 neurons and bursting pyramidal neurons [44]. CA1 neurons displayed no alterations in mAHP following apamin treatment, whereas bursting pyramidal neurons in the subiculum showed reductions in Na^+^ driven, mAHP following apamin [44].

The mode of action of SK channels in regulating neuronal excitability is the production of an outward K^+^ current in response to a depolarizing stimulus, thus contributing to returning the neuron to a more hyperpolarized membrane potential. The SK currents in the HS group are reduced compared to NS and as such, the accumulation of intracellular K^+^ in the HS group results in a reduction in the ability to reduce excitability. When examining the stimulus-response slope, NS averages ~0.1, which is significantly increased to ~0.25 in NS with apamin. HS treatment drastically increased the baseline slope to ~0.2 compared to NS ~0.1 and HS with apamin to ~0.28. Since SK currents are significantly reduced in HS versus NS, ~12 pA versus ~40 pA, respectively, we attribute the increased sensitivity to a stimulus response in HS is at least in part due to reduction in SK currents. NS with apamin versus HS with apamin had no statistical difference in their slopes to graded increases in current injection. This finding is supportive of our interpretation of loss of SK channel function in PVN neurons in rats with HS intake increases neuronal sensitivity to a depolarizing stimulus, as blockade of SK channels in NS or HS groups with apamin has nearly identical slopes (Figure 3(c)).

SFA is defined as when stimulated with depolarizing square pulse, neurons show a reduction in the firing frequency following an initial increase. This loss of SFA is usually at least in part as a result of decreased ISI [20]. When examining the raw traces to a +200 pA current injection in NS control cells, SFA is clearly visible as the interval between action potentials gradually lengthens (Figure 3(a), black). This ability is less pronounced in HS control cells (Figure 3(a), blue) and is completely absent in NS (Figure 3(a), red) and HS (Figure 3(a), purple) neurons treated with apamin. When examining the slope of the ISI (Figure 4(b)), one can note a reduction in the slope between NS and HS, ~2.0 versus ~1.1, respectively. We interpret the reduced slope of the ISI in the HS group compared to NS control as at least partially due to the loss in SK channel function. Supportive of this interpretation is that blockade of SK channels significantly reduced the slope of the ISI in both NS controls and the HS group and almost abolished the difference of the ISI between NS controls and the HS group. This suggests that the reduced SK channel function contributes at least partially to decreased ISI and loss of SFA in PVN-RVLM neurons in rats with HS intake.

### 4.1. Perspective

HS intake is a major risk factor which can contribute to the development of cardiovascular disease. While HS alone may not lead to the clinical manifestations of hypertension, it is capable of altering intrinsic properties of neurons especially those with regulation of sympatoexcitation. The present study evaluated the effects of HS intake on SK currents and neuronal excitability among PVN-RVLM neurons and revealed a reduction in SK channel function and increased PVN-RVLM neuronal excitability in HS diet-fed animals. We could expect that some factors including exercise training upregulate SK channel function expressed in the PVN, in turn, reduce sympathetic outflow and arterial blood pressure in SSH or spontaneous hypertension. SK channels could be a future target for treating salt-retaining cardiovascular disease including SSH, and congestive heart failure remains a topic that needs to be explored.

## Figures and Tables

**Table 1:** Properties of PVN-RVLM neurons.

Group	*n*	*V*_m_ (mV)	*C*_m_ (pF)	*R*_input_ (GΩ)	*V*_t_ (mV)
NS	6	−57.8 ± 3.2	51.2 ± 4.7	0.54 ± 0.03	−42.8 ± 2.8
NS apamin	5	−55.8 ± 1.7	44.8 ± 3.3	0.78 ± 0.11	−38.0 ± 1.3
HS	6	−59.3 ± 1.5	47.0 ± 4.3	0.83 ± 0.08	−40.4 ± 1.8
HS apamin	6	−58.0 ± 1.0	41.2 ± 2.4	0.96 ± 0.11	−41.0 ± 2.1

*V*_m_: resting membrane potential; *C*_m_: membrane capacitance; *R*_input_: depolarizing input resistance; *V*_t_: subthreshold of membrane potential to fire action potential.
